# Health Related Quality of Life in Patients with Onco-hematological Diseases

**DOI:** 10.2174/1745017902016010174

**Published:** 2020-07-30

**Authors:** Giorgio La Nasa, Giovanni Caocci, Emanuela Morelli, Elena Massa, Antonio Farci, Laura Deiana, Elisa Pintus, Mario Scartozzi, Federica Sancassiani

**Affiliations:** 1 Ematologia e CTMO, Ospedale Businco, Azienda Ospedaliera Brotzu, Cagliari, Italy; 2 Dipartimento di Scienze Mediche e Sanita Pubblica, Universita di Cagliari, Cagliari, Italy

**Keywords:** HRQoL, Hematological cancer, Mood depression, SF-12, Multiple Sclerosis, Tumors, Celiac disease

## Abstract

**Background:**

HRQoL is generally conceptualized as a broad multidimensional construct that refers to patients' perceptions of the impact of disease and its treatment on their physical, psychological, and social functioning and well-being. Little is known in patients with onco-hematological cancer in comparison with the general population and other chronic diseases.

**Objective:**

We assessed HRQoL in patients diagnosed with haematological cancers in comparison with the general population and other chronic diseases.

**Methods:**

The questionnaire Short Form (SF)-12 was administered to 62 patients with onco-hematological disease and results were compared with 702 controls (184 healthy people, 37 Major Depression, 201 Multiple Sclerosis; 23 Wilson disease; 46 Carotidal Atherosclerosis; 60 Celiac disease; 151 solid tumours).

**Results:**

HRQoL in patients diagnosed with a haematological cancer was significantly worse in comparison with the general population (F= 43.853, p <0.00001) but similar when compared with solid tumour and other chronic diseases such as Major Depression and Carotid Atherosclerosis. In addition, HRQoL in patients diagnosed with a haematological cancer was significantly higher than that due to Celiac disease (p <0.00001) and Wilson's disease (p= 0.02), and lower than that due to Multiple Sclerosis (p= 0.032).

**Conclusion:**

This study confirmed that haematological cancers negatively affects overall HRQoL. The results showed an impact of haematological cancers on HRQoL that is similar to what found in patients with solid tumors, Major Depression and Carotid Atherosclerosis. Current successful therapeutic strategy achieved in the treatment of haematological cancers not only positively impact on survival rate but also could improve the overall HRQoL.

## 1 INTRODUCTION

Cancer is the second leading cause of death in Italy, second only to cardiovascular disease [1]. Nonetheless, in 2017 survival has been consistently increasing in Italy after the diagnosis of cancer, specifically 3.304.648 people out of a population of around 60 million [1]. Early detection, screening, and diagnosis have demonstrated to significantly improve patient survival rates and also increase awareness of the benefit of prompt therapies and healthy lifestyles. In this context, it emerges the joint relevance of the health related quality of life (HRQoL) as a prognostic factor of clinical outcome and adherence to treatment [2-4].

HRQoL is a complex, multifaceted construct that includes' illness perception and self-evaluation of physical, mental, and social health status of the individuals [5]. HRQoL in patients with cancer is considered a critical prognostic factor for predicting survival, regardless of the clinical and socio-demographic characteristics well-known to have an important impact on the progress of the disease [6, 7]. Moreover, clinicians and patients now frequently face difficult choices concerning therapies that are often comparable in regard to efficacy, especially making medical complex decisions . In addition, cancer treatments can affect several dimensions of quality of life: body image, fatigue and mental health [8].

So far, little attention has been paid to the effects on HRQoL in patients diagnosed with haematological cancer [9-13]. Life expectancy in patients with haematological cancer is generally increased and sometimes is now close to that observed in the general population for all ages [14]. In particular circumstances, therapy can be suspended and patients can be monitored during the phase of remission free of treatment [15, 16]. In this context, the application of patient-reported outcomes (PRO), an instrument that evaluates health outcomes from the patient's perspective, plays a crucial role [17-21].

The primary endpoint of our research was to assess self-perceived HRQoL in patients diagnosed with haematological cancers and compare the results with the general population and with different chronic pathologies.

## 2 MATERIALS AND METHODS

### 2.1 Sample and Setting

Patients were recruited during the period of June-July 2018 from the Day-Hospital Service (DH) of the Hematology Unit and Stem Cell Transplantation Center, Hospital Businco, Azienda Ospedaliera Brotzu, Cagliari, Italy. The study population included patients receiving active treatment with age >18 years, both sexes and histologic confirmation of malignant neoplasm.The study was approved by the ethics committee of the Italian National Health Institute, Rome ("Istituto Superiore della Sanita"). All procedures were carried out in accordance with the 1964 Helsinki declaration and its later amendments. Each participant was provided with full descriptions of the aims and procedures of the study before signing the informed consent form. Participants were also made aware of data protection and ensured that they could terminate the interview at any time.

### 2.2 Questionnaire

The questionnaire SF-12 (Short Form Health Survey - 12 item) was administered to evaluate the HRQoL [22]. The instrument consisting of 12 items was used to investigate the two sub-dimensions, physical and psychosocial health. The total score (range: 12-47 points) consisted of asking subjects to indicate the extent to which they agreed or disagreed with individual items. The resulting answers were then scored to obtain a Likert Scale value. Higher scores reflect a better perceived HRQoL; a score <36 indicates low perceived HRQoL levels [22].

In addition, data were collected through a socio-demographic and clinical-oncological data collection form, countenancing information on marital and employment status, education level, time of follow-up, cancer staging, toxicity of treatments, intent of treatment, response and adherence to treatment. The diffusion of hematological cancer was scored from 1 to 4 considering 1 as a unique localization in one nodal station or extra-nodal; 2 as two or more localizations from the same side of diaphragm, 3 as localizations from both side of diaphragm and 4 diffuse disease. The toxicity of treatment was scored from 1 (mild) to 5 (death), according to Common Toxicities Criteria (CTC), version 4.0 [23].

### 2.3 Statistical Analysis

For each individual with onco-haematological disease, four healthy control subjects matched by gender and age were extracted from a database of a community survey on well-being and mental health that used a similar methodology [24]. The Control Sample (CS) extracted from the general population, through simple random sampling and matched for age and gender, consists of 88 females and 160 males for a total of 248 people. The impact of the pathology on the HRQoL was calculated by the difference between the mean SF-12 score obtained in the group of patients in comparison with HRQoL scores obtained from the healthy general population. Data on different chronic diseases coming from previously published studies were used for comparison [25-31].

The scores obtained in the SF-12 scales were compared using analysis of variance (ANOVA)test. Differences with a p-value of less than 0.05 were considered statistically significant.

## 3 RESULTS

The Study Sample (SS) consists of 62 people, including 22 females (35.5%) and 40 males (65.5%). The socio-demographic and clinical-oncological characteristics of the study sample are illustrated in Table **1**. The mean age of the sample for the SS was 57.23±16.5 years and 57.40±15.5 years for the CS. In the SS the average of the SF-12 total score was 32.60±5.43; in the CS the average of the SF-12 total score is 38.04±5.87. HRQoL in patients diagnosed with a haematological cancer was found significantly worse in comparison with the general population (F= 43.853, p <0.00001, df 1, 308.309). The impact on HRQoL of the burden attributable to the cancer was measured in 5.44 ± 2.93 points on the SF-12 scale without differences between genders(5.19±3.18 in females and 5.53±3.02 in males, p=0.6).

The HRQoL profile in patients diagnosed with a haematological cancer was comparable to that with solid tumour (F = 0.770; df= 1,211,212; p= 0.381) and other chronic diseases such as Major Depression (F= 0.255; df= 1.95.96; p= 0.615), and Carotid Atherosclerosis (F= 0.115; df= 1.106.107; p= 0.735). The impact on HRQoL was significantly greater than that due to Celiac disease (F= 67.959; df= 1.120.121; p <0.00001) and Wilson's disease (F= 5.623; df= 1.83.84; p= 0.02) and significantly lower than that due to Multiple Sclerosis (F= 4,673; df= 1,261.9262; p= 0.032) (Table **2**).

## 4 DISCUSSION

This study suggests that patients with haematological cancers have significantly worse HRQoL outcomes than their peers in the general population [9, 10, 24]. Results are confirmed in males and females although it is recognized that within the general population women have poorer HRQoL [24]. The results of our study showed that both haematological and solid cancers have a similar impact on the patient HRQoL, along with other chronic diseases such as Major Depression, and Carotid Atherosclerosis; a significantly higher impact of haematological cancers on HRQoL was reported compared with Celiac and Wilson's disease but lower compared with Multiple Sclerosis [26-28].

In this regard, it must be taken into consideration that the "raw" score on the respective SF-12 scale is comparable among cases and controls but not among pathologies since the different gender and age distribution as well as other risk factors, such as schooling and dissimilar comorbid conditions can have an impact on the outcomes. For example, atherosclerosis is classed as a disease of aging, such that increasing age is a risk factor for the development of atherosclerosis. Nonetheless, comparing SF12 scores across different diseases and the respective age and gender controls scores could be considered correct.

It is thus important to take into account all comorbidities and their relative effect on quality of life state when we consider specific conditions. It is also important to ensure an accurate calculation of the "Attributable Burden" of a specific diagnosis. An example is that about 30% of patients with cardiovascular diseases experience an excess of mood disturbances [24]. Instead, if these conditions are not typically related to the diagnosis but intrinsic to the population characteristics, therefore independent from the diagnosis itself, the effect is cancelled by matching gender and age variables. The calculation of the burden of the disease allows a comparison across different pathologies.

In this respect, we reported that in a sample of patients with haematological cancers, in which the vast majority have a course disease of more than one year and advanced stage, the patients share the same HRQoL profile as in the case of other chronic diseases. Interestingly, lower HRQoL was reported when compared with patients with multiple sclerosis although patients with haematological cancers experience a prolonged expectancy of life. This indicates that the current therapeutic regime to treat haematological cancers not only lead to a better survival rate but also improve acceptance and quality of life.

This study has limitations. One limitation is relative to the observational nature of cross-sectional study that does not permit estimating the perceived HRQoL differences over time as instead longitudinal study would permit. Also, the small sample size decreases the power of the difference between HRQoL and both socio-demographic and clinical-oncological anamnestic variables. Furthermore, SF-12 questionnaire, as health generic scale, may not express adequately the changes across different pathological conditions examined in this study. Nevertheless, considering the paucity of HRQoL reports in hematological malignancies, this study contributes to new insight in this field.

## CONCLUSION

This study suggests that haematological cancers negatively affect overall HRQoL. The patients diagnosed with haematological cancer have poorer perceived HRQoL than their peers in the general population.

The results showed an impact of haematological cancers on HRQoL that is similar to what found in patients with solid tumors, Major Depression and Carotid Atherosclerosis. Differently, the impact of haematological cancer on HRQoL compared with other strongly disabling disorders such as multiple sclerosis was reported to be lower.

Current successful therapeutic strategy achieved in the treatment of haematological cancers not only positively impacts on survival rate but could also improve the overall HRQoL.

## Figures and Tables

**Table 1:** **Sociodemographic and clinical characteristics of patients with onco-hematological disease involved in the study.** The diffusion of hematological cancer was scored from 1 to 4 considering 1 as a unique localization in one nodal station or extra-nodal; 2 as two or more localizations from the same side of diaphragm, 3 as localizations from both side of diaphragm and 4 diffuse disease. The toxicity of treatment was scored from 1 (mild) to 5 (death), according to common toxicities criteria (CTC), version 4.0 *data not available

**VARIABLES**		**(N°)**	**(%)**
**Marital status**	Single	17	27.4
	Married/Living with partner	41	66.1
	Divorced	1	1.6
	Widow	3	4.8
**Employment status**	Housewife	6	9.7
	Unemployed	5	8.1
	Employed	19	30.6
	Retired	29	46.7
	Student	3	4.8
**Education level**	Primary school	4	6.5
	Secondary school	24	38.7
	High school	24	38.7
	University degree	8	12.9
	Higher	2	3.2
**Outpatient service**	Outpatient	62	78,2
**Follow-up**	First admission	1	1.6
	<6 months	7	11.3
	6-12 months	7	11.3
	>12 months	46	74.2
	NA*	1	1.6
**Cancer stage**	1	1	1.6
	2	7	11.3
	3	6	9.7
	4	44	71
	NA*	4	6.5
**Toxicity of treatments**	1	1	1.6
	2	5	8.1
	3	14	22.6
	4	25	40.3
	5	13	21
	NA*	4	6.5
**Intent of treatment**	Curative	31	50
	maintenance	22	35.5
	Supportive	5	8.1
	NA*	4	6.5
**Response to treatment**	Complete response	6	9.7
	Partial response	27	43.5
	Progression	3	4.8
	Ongoing evaluation	22	35.5
	NA*	4	6.5
**Adherenceat 3 months of follow-up**	Yes	54	87.1
	No	1	1.6
	Not evaluated	3	4.8
	NA*	4	6.5

**Table 2:** Attributable burden on HRQoL in patients with onco-hematological disease: comparison with chronic tumors or diseases.

**Disease**	**SF-12 score** **Average±sd**	**Attributable Burden on HRQoL**	**Comparison with Onco-hematologic Disease** **ANOVA 1 way**	**p**
Major Depression (N=37)	33.8±9.2	5.6±3.6	df 1,95,96 F=0.255	0.615
Multiple Sclerosis (N=201)	29.5± 7.3	7.0±3.5	df 1,261,9262 F=4.673	**0.032**
Wilson Disease (N=23)	33.8±9.0	4.4±1.7	df 1,83,84 F=5.623	**0.020**
Carotidal atherosclerosis (N=46)	30.6±8.1	6.2±5.0	df 1,106,107 F=0.115	0.735
Celiac disease (N=60)	35.83±5.72	2.4±1.0	df 1,120,121 F= 67.959	**<0.00001**
Solid tumors (N=151)	32.34±6.764	4.67±6.64	df 1,211,212 F= 0.770	0.381
Onco-hematological tumors (N=62)	32.60±5.43	5.44±2.93		

## LIST OF ABBREVIATIONS

HRQoL: = Health Related Quality of Life
SS: = Study Sample
CS: = Control Sample
CTX: = Common Toxicities Criteria
